# A Rapid, Sensitive, and Portable Biosensor Assay for the Detection of Botulinum Neurotoxin Serotype A in Complex Food Matrices

**DOI:** 10.3390/toxins10110476

**Published:** 2018-11-15

**Authors:** Christina C. Tam, Andrew R. Flannery, Luisa W. Cheng

**Affiliations:** 1 Foodborne Toxin Detection and Prevention Research Unit, Western Regional Research Center, Agricultural Research Services, United States Department of Agriculture, 800 Buchanan Street, Albany, CA 94710, USA; christina.tam@ars.usda.gov; 2 PathSensors, Inc. 701 East Pratt Street, Baltimore, MD 21202, USA; aflannery@pathsensors.com

**Keywords:** botulinum neurotoxin, biosensor, CANARY^®^, detection, B-cell based assay, immunoassay, food matrices

## Abstract

Botulinum neurotoxin (BoNT) intoxication can lead to the disease botulism, characterized by flaccid muscle paralysis that can cause respiratory failure and death. Due to the significant morbidity and mortality costs associated with BoNTs high toxicity, developing highly sensitive, rapid, and field-deployable assays are critically important to protect the nation’s food supply against either accidental or intentional contamination. We report here that the B-cell based biosensor assay CANARY^®^ (Cellular Analysis and Notification of Antigen Risks and Yields) Zephyr detects BoNT/A holotoxin at limits of detection (LOD) of 10.0 ± 2.5 ng/mL in assay buffer. Milk matrices (whole milk, 2% milk and non-fat milk) with BoNT/A holotoxin were detected at similar levels (7.4–7.9 ng/mL). BoNT/A complex was positive in carrot, orange, and apple juices at LODs of 32.5–75.0 ng/mL. The detection of BoNT/A complex in solid complex foods (ground beef, smoked salmon, green bean baby puree) ranged from 14.8 ng/mL to 62.5 ng/mL. Detection of BoNT/A complex in the viscous liquid egg matrix required dilution in assay buffer and gave a LOD of 171.9 ± 64.7 ng/mL. These results show that the CANARY^®^ Zephyr assay can be a highly useful qualitative tool in environmental and food safety surveillance programs.

## 1. Introduction

*Clostridium* spp. are ubiquitous, gram-positive, anaerobic spore-forming microorganisms that express some of the most potent neurotoxins known to man. Botulinum neurotoxins (BoNTs) cause botulism, which is distinguished by flaccid muscle paralysis [1,2]. There are several antigenically and serologically distinct serotypes (A–H); currently, BoNT serotypes A, B, E, and F are known to cause disease in humans [3,4,5,6,7]. These neurotoxins are a public health and safety threat due to their highly toxic nature with a parenteral lethal dose of 0.1–1 ng/kg and with an estimated oral intoxication dose of 1 μg/kg. The significant morbidity and mortality associated with such doses of botulinum neurotoxin intoxication necessitates the development of a field-deployable assay capable of detecting toxins at a high sensitivity and specificity that also is compatible with food and environmental samples. Such diagnostics will allow for both the clinical identification of intoxication and the surveillance of consumables for adulteration as a means to start treatment and dispose of contaminated resources.

There are numerous methods (in vivo, ex vivo, and in vitro) that are currently used to detect botulinum neurotoxins and/or *C. botulinum* contamination. The in vivo mouse bioassay is considered the “gold standard” because of its high sensitivity (limits of detection (LOD) ≅ 20.0–30.0 pg) [8,9] and reliability to model all aspects of BoNT intoxication [10,11]. However, this assay is time-consuming, expensive, and requires experienced personnel and specialized facilities. Additionally, the in vivo toe spread reflex model has been tested for the detection of BoNT in buffer, serum, and milk [10]. Alternative ex vivo animal assays, such as the mouse phrenic nerve hemidiaphragm assay, have been developed and are sensitive and faster than the mouse bioassay, but even such alternatives require special equipment and personnel—and they are not compatible for use with complex matrices.

In addition to the in vivo and ex vivo models described above, a plethora of in vitro assays also have been developed and described in the literature. These assays may be divided into seven different categories: (1) immunological and antibody-based assays; (2) nucleic acid-based assays; (3) lateral flow methods; (4) mass-spectrometry based methods; (5) enzymatic based assays; (6) cell-based assays; and (7) antibody and biosensor technologies. Some well-known in vitro assays are ELISA, ECL, lateral flow, ENDOPEP-MS, ENDOPEP-ELISA, Spin-Dx, Immuno-PCR, ALISSA, SNAPtide, VAMPtide, and SYNTAXtide. Additionally, newer assays have combined different technologies to improve the sensitivity of detection. Depending on the assay, the detection limits range from sub-picogram to nanogram per mL or attomolar to pM for buffer and some food matrices [12,13,14,15,16,17,18,19,20,21,22,23,24,25,26,27,28,29,30,31].

CANARY^®^ (Cellular Analysis and Notification of Antigen Risks and Yields) is a cell-based biosensor technology. The technology relies on immortal B-cell lines that express antibodies that are specific to a target and also contain aequroin, a calcium-sensitive bioluminescent protein from the *Aequoria victoria* jellyfish. Initial work with CANARY^®^ technology resulted in the detection of pathogens such as *Yersinia pestis*, Vaccinia virus, Venezuelan equine encephalitis virus, *E. coli* O157:H7, and *Bacillus anthracis* with specificity, high sensitivity, rapidity, and small volumes [32]. Recently, the CANARY^®^ Zephyr system was evaluated against a variety of immunoassays (mostly lateral flow) as well as other biological indicator tests using the potential bioterror threats *Bacillus anthracis* and ricin. The study found that the limit of detection of ricin was 3.0 ng/mL and 10^3^ spores/mL for *B.anthracis* [33]. The authors found that compared against various commercially available kits, the CANARY^®^ Zephyr platform was 4 orders of magnitude more sensitive for detecting *B. anthracis* and was the most sensitive for ricin.

Though there are multiple technologies that are used to detect botulinum neurotoxins in buffer and complex matrices, each of the technologies have their strengths and weaknesses. The negatives may be due to the time required for experimentation, cost, expert personnel, specialized facilities, expensive and bulky equipment, sensitivity, or incompatibility with complex matrices such as sera, milk, juices, ground meat, eggs, and smoked fish. Therefore, the reality is that multiple technologies may be required for specific conditions to make rapid determinations, especially in food safety and environmental settings. In this study, we sought to evaluate the feasibility of using the CANARY^®^ Zephyr system to detect botulinum neurotoxin serotype A in buffer as well as in 10 complex matrices. Qualitative determination of relative limits of detection and specific sample preparation protocols will be determined.

## 2. Results

### 2.1. CANARY^®^ Zephry B-Cell Based Assay Can Detect Botulinum Neurotoxin Serotype A with High Sensitivity

**Figure 1:**
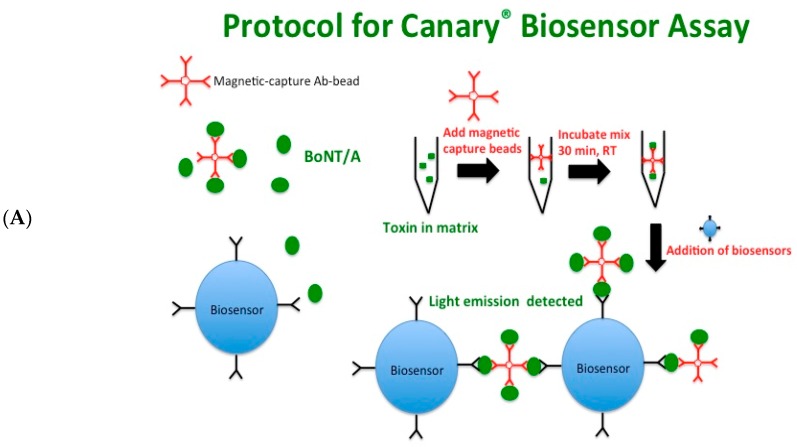

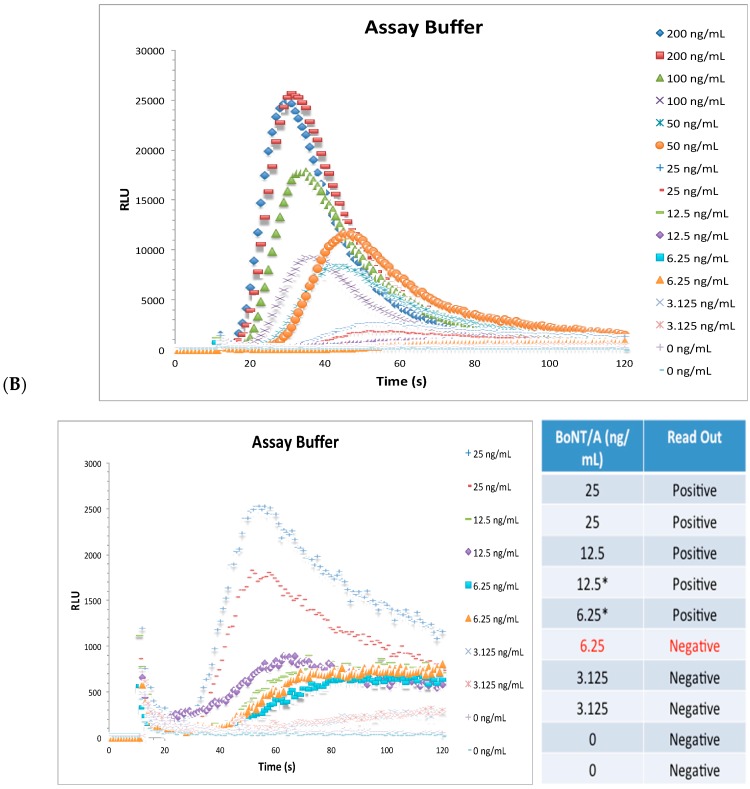
CANARY^®^ biosensor assay detects BoNT/A holotoxin in assay buffer in a concentration-dependent manner. (**A**) Schematic of CANARY^®^ biosensor assay. (**B**) The top graph shows a representative graph depicting the relative light unit (RLU) detected by a luminometer as the concentration of the BoNT/A holotoxin: immunomagnetic bead complex is bound to the biosensors. The bottom graph shows an inset of the graph showing the RLU from 25 ng/mL to 0 ng/mL of BoNT/A holotoxin. The adjacent table shows the read out generated from the CANARY^®^ biosensor assay. To calculate the limit of detection of this experiment, the last two positive readings (*) were used. The red text indicates the first negative reading (one duplicate of 6.25 ng/mL). These are representative graphs from one independent experiment. 11 μL samples were used for the biosensor assay A total of *n* = 8 independent experiments with duplicates were used to calculate the final limit of detection of BoNT/A holotoxin in assay buffer.

Figure 1A depicts a schematic of the CANARY^®^ biosensor assay. Immunomagnetic capture beads specific to BoNT/A were incubated with toxin in buffer or matrix for 30 min at room temperature to allow for the toxin:immunomagnetic bead complex to form a multi-valent epitope. Biosensors expressing membrane-bound antibodies that are specific to a different epitope of BoNT/A than those used on the magnetic beads were then added to the reaction. The binding of the multi-valent epitope on the magnetic beads by the antibodies on the biosensors’ surface leads to antibody clustering or “crosslinking”, which results in an intracellular calcium influx that activates the aequorin molecules and, hence, luminescence. The luminometer detects the light output, which is expressed as relative light units (RLU) over time (120 s, read every second).

Assay sensitivity for BoNT/A was first determined in buffer provided by the manufacturer. Serial dilutions of toxin in assay buffer were made and the luminescent signal was measured for each reaction in duplicate. Figure 1B shows that there is a good sensitivity for BoNT/A and the relative light unit detected (RLU) is concentration-dependent. The Zephyr software depicts the RLU detected by the luminometer as a graph in real-time (Figure 1B, top and bottom graphs) and then the sample is determined to be either positive or negative based on a proprietary algorithm based on a combination of signal-to-noise ratio and up to 28 different coefficients to determine positive and negative curve characteristics (Figure 1B, bottom table). As one can determine from this experiment, one of the 6.25 ng/mL samples was positive (Figure 1B, bottom graph, orange) for BoNT/A holotoxin while the other duplicate (Figure 1B, bottom graph, teal) was not even though there was a similar but slightly less RLU above the zero toxin buffer controls (Figure 1B, bottom graph, table). The 3.125 ng/mL duplicates both had signal above the toxin controls but were both considered negative according to the threshold calculated from the Zephyr software. Therefore, the LOD from this experiment would be the average of 12.5 ng/mL and 6.25 ng/mL. Additionally, the CANARY^®^ biosensor assay is specific for BoNT/A in a comparison with BoNT/F at 1.0 mg/mL (Figure S1).

### 2.2. Zephyr Detects BoNT/A in Whole Milk, 2% Milk, and Non-Fat Milk

**Figure 2:**
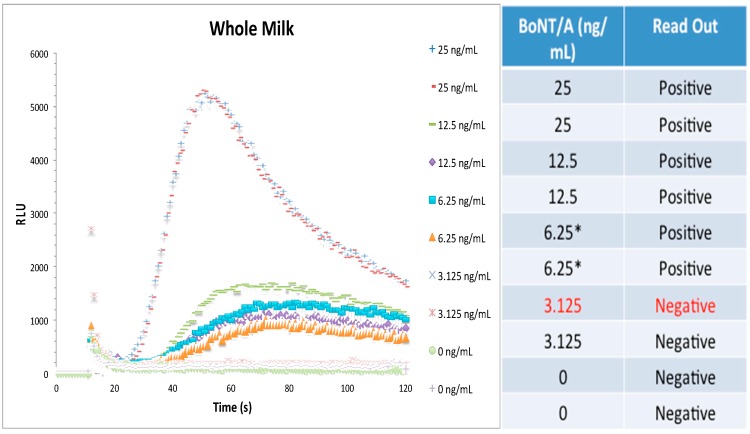
Whole milk has no effect on the detection of BoNT/A using CANARY^®^. A toxin concentration-dependent signal similar to assay buffer was detected in the whole milk matrix. The graph shows the RLU from 25 ng/mL to 0 ng/mL of BoNT/A holotoxin in whole milk from one representative experiment was used. To calculate the limit of detection, the last two positive readings (*) were used. The red text indicates the first negative reading (3.125 ng/mL). For this experiment, both duplicates at 6.25 ng/mL were positive. This is one representative graph from one independent experiment with duplicates. 11 μL samples were used for the biosensor assay. A total of *n* = 6 independent experiments in duplicates were used to calculate the final limit of detection of BoNT/A holotoxin in whole milk.

Based on reports with other toxins, one would expect the detection of BoNT/A in assay buffer with a high level of sensitivity. However, detection assays should be flexible enough to detect BoNT/A in various complex matrices that may be from food and environmental settings, since these would be the types of samples that would be evaluated from governmental, diagnostic, and pharmaceutical laboratories. Therefore, three different milk matrices (whole milk, 2% milk, and non-fat milk) were spiked with holotoxin and serially diluted in matrix; then, the biosensor assay proceeded in the same fashion as for assay buffer. Figure 2 shows the live graph results using whole milk as well as the table read-out. Similar to assay buffer, RLU is concentration-dependent and the LOD was 6.2 ng/mL from this one experiment.

Table 1 shows the estimated limits of detection for the CANARY^®^ assay for BoNT/A holotoxin in assay buffer and the three milk matrices. The limit of detection was determined to be the average of the last two positive samples by the Zephyr program using a proprietary algorithm based upon previous work and refined by PathSensors, Inc. [32]. BoNT/A holotoxin LOD in assay buffer was 10.0 ± 2.5 ng/mL from *n* = 8 independent experiments in duplicates (Supplemental Figure S2). Whole milk, 2% reduced milk, and non-fat milk had LODS of 7.4 ± 2.2 ng/mL, 7.9 ± 2.5 ng/mL, and 7.6 ± 2.3 ng/mL from *n* = 6 independent experiments in duplicates (Figure S2).

### 2.3. Detection of BoNT/A in Acidified Juices Requires Neutralization

**Figure 3:**
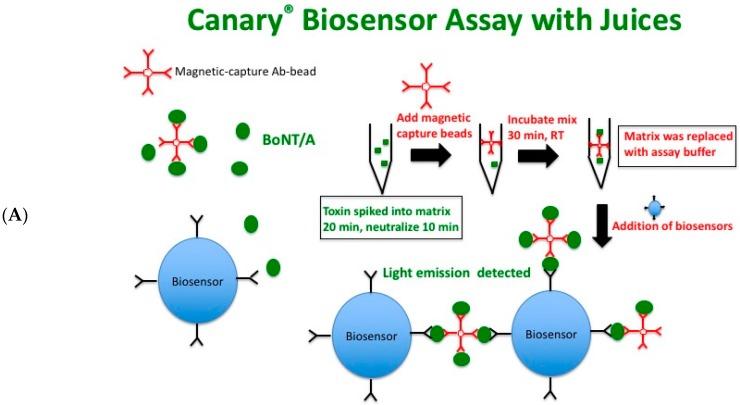

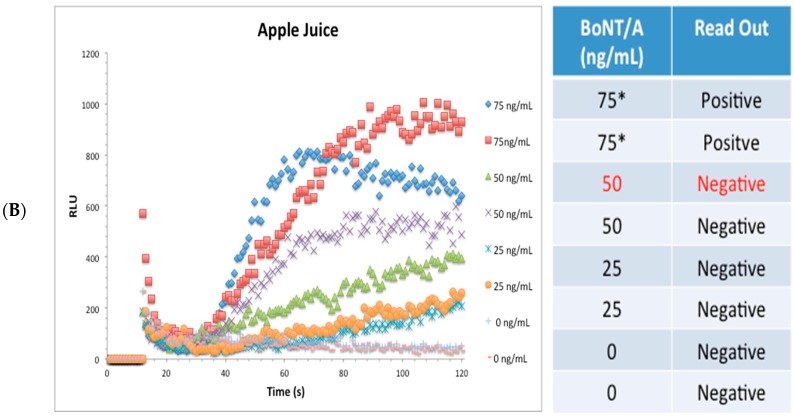
Acidic juices require neutralization before usage with the CANARY^®^ biosensor assay. (**A**) Schematic of the CANARY^®^ biosensor assay used with acidic juices. BoNT/A complex was spiked into the acidic juices at various concentrations for 20 min at room temperature. The spiked juices were neutralized with 10% 5 M Tris pH 8.0 for 10 min before addition of magnetic capture beads. After binding of capture beads, a magnetic bead separator was used to capture the toxin: immunomagnetic bead complex to remove matrix and then replacement with an equal volume of assay buffer. (**B**) A toxin concentration-dependent increase in signal is detected. 50 μL samples were used for the biosensor assay. Five independent experiments in duplicates were performed and one representative data set is presented.

Acidic juices such as apple, carrot, and orange have been used as liquid matrices for study by many BoNT detection platforms. We wanted to validate the use of the biosensor assay against these three commonly tested matrices. A schematic of the CANARY^®^ biosensor assay protocol for acidic juices is shown in Figure 3A. Acidic juices were first spiked with BoNT/A complex for 20 min at room temperature and then neutralized with 5 M Tris pH 8.0 (10% final volume) for 10 min before adding magnetic capture beads. After incubation with the capture beads, a magnetic separator was used to capture the toxin matrix:immunomagnetic bead complex; removal of matrix, and then replacement with an equal volume of assay buffer. Biosensors were then added and luminescent signal was captured. As seen in Figure 3B, detection of BoNT/A complex in apple juice was possible but, as compared to assay buffer and milk matrices, the sensitivity (75.0 ± 21.6 ng/mL) was decreased.

Table 2 shows the estimated limits of detection for the CANARY^®^ assay in the detection of BoNT/A complex in spiked and then neutralized orange, apple, and carrot juices. The three juice matrices had higher limits of detection than assay buffer or the three milk matrices. Carrot juice had a LOD of 32.5 ± 12.0 ng/mL (Figure S3) which was better than orange juice (62.5 ± 21.2 ng/mL) and apple juice (75.0 ± 21.6 ng/mL) from *n* = 5 independent experiments in duplicates.

### 2.4. Detection of BoNT/A in Liquid Egg, Ground Beef, Green Bean Baby Food, and Smoked Salmon

Since the biosensor assay can detect BoNT/A in assay buffer, milk matrices, and acidic juices with varying levels of sensitivity, more complex matrices consisting of liquid egg, ground beef, green bean baby food, and smoked salmon were spiked with toxin and the LOD for each complex matrix was determined. The estimated limits of detection for the CANARY^®^ assay in the detection BoNT/A complex in liquid egg, ground beef, green bean baby puree, and smoked salmon are presented in Table 3. These complex matrices required minor modifications to the biosensor assay in terms of sample preparation. Liquid egg (egg yolk and white) was very viscous and attempts were unsuccessful in detecting BoNT/A complex using the biosensor assay. Therefore, liquid egg was diluted 1:10 into assay buffer and toxin was spiked into each tube of the 1:10 liquid egg/assay buffer mixture with specific concentrations instead of serial dilution. Solid complex food extractions were guided by previous studies as well as standard extraction methodologies used by regulatory agencies [22,31,34]. A mass of 0.025 g of ground beef, green bean baby food, and smoked salmon were weighed out and put in reaction tubes with specified toxin doses and assay buffer to a volume of 250 μL. The samples were incubated at room temperature for 30 min before centrifugation at 10,000× *g* for 5 min. Then 50 μL of the cleared supernatant was used with the magnetic capture beads for the continuation of the assay. Amongst these matrices, liquid egg diluted 1:10 matrix gave the lowest sensitivity of 171.9 ± 64.7 ng/mL (Figure S4); it is at this concentration after a 1:10 dilution indicating that the concentration of BoNT/A actually present in the undiluted matrix is much higher (≈2 μg/mL). BoNT/A was detected by the assay in both ground beef (14.8 ± 2.6 ng/mL) (Figure S4) and green bean baby food (16.6 ± 6.5 ng/mL) (Figure S5), thus indicating that these two types of matrices are amenable to the assay). Smoked salmon had the lowest level of sensitivity of the three solid complex foods (62.5 ± 0.0 ng/mL) (Figure S5).

## 3. Discussion

BoNTs are among the most poisonous substances known to man and cause the disease botulism, which is distinguished by flaccid muscle paralysis that can lead to respiratory failure and death. Botulism occurs through three routes: foodborne, wound, and infant botulism. BoNTs are considered Tier 1 Select Agents (CDC) and pose a public health and food safety concern due to the their potential use by bioterrorists. Assays to detect BoNTs must have high sensitivity and specificity, be compatible with food and environmental samples, and they also should be field-deployable to be of use to a variety of people including first-responders, diagnostic technicians, and food inspectors.

BoNT detection assays utilize multiple methods including antibody-based, mass-spectrometry, nucleic acid-based, cell-based, and enzymatic assays, as well as in vivo and ex vivo mouse assays in buffer and some matrices [8,9,10,11,12,13,14,15,16,17,18,19,20,21,22,23,24,25,26,27,28,29]. All of the current technologies have advantages and disadvantages. Advantages are: high sensitivity, faster time, cost-effectiveness, smaller volumes, complex sample compatibility, and multiplex capability. However, the disadvantages are notable and include long experiment times; high costs associated with testing, including the need for expensive (and unwieldy) equipment; the need for expert personnel to conduct the experiments and specialized facilities in which to conduct them; and sensitivity or incompatibility with complex matrices such as sera, milk, juices, ground meat, eggs, and smoked fish. No single technology is adequate for use in food safety or environmental settings; thus, all available and new technology platforms should be assessed for their ability to detect BoNTs.

The CANARY^®^ Zephyr system utilizes a B-cell based biosensor system coupled with immunoprecipation of the toxin complex to detect BoNT/A. This study has found that the assay is rapid: (<40 min) from the addition of immunomagnetic capture beads to the reaction matrix to the read out of luminescence and final qualitative determination of positive or negative for the sample tested. The sample preparation time is variable depending on the type of sample matrix and this will add more time to any assay. The CANARY^®^ Zephyr system is also portable, deployable to potentially manufacturing or processing centers because it consists of a laptop, a small centrifuge, and a small luminometer that can fit in a suitcase but still fit on a small bench top space [33]. Yet, its portability does not sacrifice utility, as the assay also uses small volumes (50 μL) to facilitate multiple sample analysis for precious samples. The purpose of the technology is to rapidly triage samples that are positive for the analyte in question and to allow confirmation of suspected samples via other methods such as the mouse bioassay, which would function, similar to the qualitative positive/negative read out of lateral flow devices (LFD).

In this study, we have established that CANARY^®^ can detect BoNT/A holotoxin with a LOD of 10.0 ± 2.5 ng/mL. This LOD is within the range of lateral flow devices but not as sensitive as other assays [12,13,14,15,16,17,18,19,20,21,22,23,24,25,26,27,28,29,30,31]. An ultrasensitive gold nanoparticle endopeptidase-based lateral flow has been developed and has a LOD ≈ 20.0 pg/mL in sera comparable to the mouse bioassay; however, this assay requires 12 h of digestion but food and beverage matrices were not evaluated [28]. Ching et al., has recently described the development of a rapid LFD that is able to detect BoNT/A and BoNT/B simultaneously with a LOD of 5.0 ng/mL for both in phosphate gelatin [13]. Milk matrices (non-fat, 2%, whole milk) have a LOD range of 7.4 ng/mL to 7.9 ng/mL without further dilution into assay buffer nor requiring defatting to achieve this sensitivity. In comparison, the BoNT/A/B LFD had LODs of 100–200 ng/mL for BoNT/A in whole milk, 1% milk, and non-fat milk but required a 10-fold dilution in double-deionized water. Defatting of the milk matrices before use in this LFD achieved sensitivities of 5.0 ng/mL (2% milk) and 10.0 ng/mL (whole, non-fat milk).

Similar to previous work with other technologies such as ELISA, ECL, and LFD [13,19], sample preparation of acidic juice (orange, apple, and carrot) required neutralization of pH for the CANARY^®^ assay. Neutralized acidic juice samples were not diluted [35] nor centrifuged to remove particulate matter as used previously with other technologies but a matrix removal step was added using a magnetic bead separator before biosensors were added. Work with BoNT/A/B LFD gave directly tested apple juice a LOD of 25.0 ng/mL that is lower than using CANARY^®^ (75 ± 21.6 ng/mL). For orange juice, the biosensor assay had a LOD of 62.5 ± 21.2 ng/mL when tested directly using the spiked orange juice. The BoNT/A/B LFD had a lower LOD (25.0 ng/mL) but their assay required a two-fold dilution into phosphate buffer before use. Regardless, the higher LODs using the biosensor assay could be due to inherent different brands of juices, pipetting, errors, toxin lots as well as the need for optimization.

Attempts to use undiluted liquid egg were unsuccessful most likely due to the inherent viscosity of the matrix inhibiting toxin binding to capture beads and/or biosensors. Therefore, liquid egg had to be diluted 1:10 with assay buffer before spiking with BoNT/A complex and subjected to CANARY^®^ giving the lowest level of sensitivity for any of the matrices (171.9 ± 64.7 ng/mL). Further optimization is required to optimize this matrix for the biosensor assay.

Solid complex foods are known to be problematic because of the particulate matter and potentially ‘fat’ content that may titrate the analyte of interest from the cleared supernatants that would be used for detection/confirmation assays. The “standard” protocol for extraction of *Clostridium* spores/bacteria and toxins used in governmental agencies is based on the FDA’s Bacteriological Analytical Manual (BAM) [34]. In this manual for *Clostridium botulinum*, one portion of solid foods are directed for detection of viable bacteria; another is removed for toxicity testing, and the rest are kept in the refrigerator. Solid foods (usually grams) are extracted with an equal volume of phosphate gelatin buffer pH 6.2 before maceration with chilled mortar and pestle (or stomacher or homogenizer). The macerated solids are centrifuged in the cold and the cleared, filtered supernatant is then used for the assays (i.e., mouse bioassay). Further treatment of the supernatant or liquid food with trypsin to activate toxins from non-proteolytic strains will need to occur before usage (1 h). Other experimental protocols have used the cleared, filtered supernatant of ground beef as the matrix to spike toxin into for detection assays [31]. However, in this study, toxin was added directly to the ground beef in assay buffer which is different than adding phosphate gelatin pH 6.2 and incubated for 30 min to allow for optimal dispersal of the toxin. This incubation step may not be necessary or the incubation time may not need to be that long but these modifications would need to be tested. After incubation, the samples were pipetted to shear the ground beef (0.025 g in 250 uL assay buffer) a few times and then vigorously vortexed for 1 min. The mixture was centrifuged to remove the particulate matter before proceeding with the biosensor assay with the cleared but non-filtered supernatant. This sample extraction protocol was applied to both green bean baby food as well as smoked salmon. The LOD of ground beef obtained was 14.8 ± 2.6 ng/mL. Green bean baby food had an LOD of 16.6 ± 6.5 ng/mL. The highest LOD from solid food matrices was smoked salmon, with a LOD of 62.5 ± 0.0 ng/mL.

From the data from the figures, supplemental figures, and data not shown; this biosensor assay in the current format has both intra-and inter-assay variations. Of importance, there are sometimes large variations in the lower range of the concentrations close to the limit of detection. Inherent to all diagnostics is the variability introduced due to different users, assay conditions, and test matrices. For the PathSensors, Inc. (PSI) BoNT/A assay, there are multiple steps: (1) binding of the antigen to capture beads; (2) recognition of the antigen by the biosensors; and (3) signal transduction and light emission. Any deviation from the prescribed protocol could affect the RLU signal as well as the assay’s performance. Common to all diagnostics, when the analyte concentration is close to the limit of detection, the variance can have a larger potential impact for missed calls. However, due to the robustness of the assay and the rigors of the algorithm design, low analyte levels are expected to give no false positives but could potentially have some false negatives. If false negatives are of concern, this assay is designed to allow for rapid retesting and the ability for users use a larger sample volume (instead of 50 µL used in the manuscript) to eliminate these concerns. Finally, optimizations in the sample processing protocol (such as additional wash steps), may help decrease the variances seen in the results described in this study, as no wash steps were included.

The original CANARY assay developed by MIT defined their limit of detection as a value that was greater than three standard deviations above the background signal. Therefore, the maximum relative luminescent unit (RLU) signal from each sample was used as the metric to determine if the sample was negative or positive for the analyte in question. However, there could be potential issues related to only using the maximum RLU signal for a given sample. For example, aberrant spikes could cause an artificially high RLU value resulting in a false positive or, alternatively, a signal with a low signal but discernable curve could yield a false negative. To minimize erroneous results, PSI developed a proprietary mathematical algorithm that analyzes the light output signals from the biosensors to differentiate a true positive curve from true negative background. This analysis was based on a mathematical algorithm first developed by MIT-LL (Massachusetts Institute of Technology: Lincoln Laboratory), but that subsequently has been refined at PSI. There are up to 28 different coefficients within this algorithm that may be set to provide filters for the classification of positive and negative assay calls. Examples of these coefficient filters include the amplitude of peak light output, the number of required upslopes in the light output prior to reaching its peak, and the start time of the first of these upslopes. By adjusting these coefficients based upon data from spiked and non-spiked material, the sensitivity and specificity of each assay can be adjusted to achieve the required performance for each analyte and matrix. Therefore, if there are deviations that cause the curve to shift then the “same” duplicate samples maybe defined differently and hence the variation seen in the lower concentration range.

In this study, the first reported study using the CANARY^®^ Zephyr biosensor assay system to detect BoNTs, we have shown that CANARY^®^ Zephyr is a useful platform that can be applicable in surveillance and environmental testing of BoNT/A with good sensitivity, short actual assay time (<40 min), amenable to different matrices, and reliable (less false positives). The critical question for all these assays is whether or not they can detect the toxin amount present in foodborne outbreaks. One report has found that 10,000 mouse lethal dose BoNT/A per gram (160 ng/g) in chili [36]. Angulo et al. reported 3.2 × 10^4^ mouse lethal dose per gram of potato dip [37]. Another foodborne outbreak had 4,000 mouse lethal dose BoNT/A per gram of hot dog chili sauce [38]. Additionally, two of the carrot juice bottles that were consumed by patients contained 100 to 100,000 (6.65 × 10^5^) mouse LD_50_ per mL (unpublished reports, J.A. and S.M) [39]. From these studies, one can definitely say that the amount of botulinum neurotoxin present in food and beverage from foodborne outbreaks is varied. Many times, botulinum neurotoxins can not be detected in suspected foods from foodborne outbreaks due to a variety of reasons. Based on the BoNT content found in recent food outbreaks, CANARY^®^ system would likely be able to detect the presence of foodborne toxins.

CANARY^®^ technology rapidly detects BoNT/A as compared to the extended time periods for other detection methods such as ELISAs (hours) and the mouse bioassay (days). Lateral flow devices, while simple and fast, have complex matrix and sensitivity issues, as well as potentially high rates of false positives in comparison to CANARY^®^ Zephyr. This assay was originally developed for “suspicious powders” and was tested in the original format and has shown to work with food matrices. Further optimization of sample extraction and biosensor protocol could yield improvements in the assay’s sensitivity. Development of new BoNT/A or utilizing known BoNT/A monoclonal antibody sequences to generate new immunomagnetic capture beads or biosensors can improve the assay. This platform could be further developed into rapid detection kits for the other BoNT serotypes and subtypes. Additionally, the company has developed new kits that can be deployed without the need for refrigeration in conjunction with current efforts to develop a single contained portable luminometer unit that can truly be field-deployable.

## 4. Materials and Methods

### 4.1. Reagents

Botulinum neurotoxin serotype A (holotoxin and toxin complex) were obtained from Metabiologics at 1 mg/mL (Madison, WI, USA). CANARY^®^ Zephyr BoNT/A kit (25 reactions) containing assay buffer, reconstitution buffer, negative control, positive control, B-cell biosensors, microcentrifuge tubes, and immunomagnetic capture beads were obtained from PathSensors, Inc. (Baltimore, MD, USA). All reagents from the kit were stored at 4 °C while the biosensors are stored in liquid nitrogen. The CANARY^®^ detection system consists of a laptop, a small microcentrifuge (SCILOGEX D1008, Rocky Hill, CT, USA), and a luminometer (Sirius L Tube Luminometer, TITERTEK-Berthold, Pforzheim, Germany). Whole milk, 2% milk, non-fat milk, orange juice (no pulp), carrot juice, apple juice, green bean baby food, 80:20% ground beef, and smoked salmon were bought from a local supermarket.

### 4.2. Biosensor Assay Protocol for Assay Buffer and Milk Matrices

Botulinum neurotoxin serotype A holotoxin at 1 mg/mL was diluted in phosphate gelatin buffer 1:100 to 10 μg/mL and allowed to disperse for 30 min at room temperature. From the 10 μg/mL stock, toxin was diluted into assay buffer or milk matrices to generate the highest concentration in a total volume of 200 μL. The sample was incubated for 30 min at room temperature before serial dilutions were made in the respective matrices. 50 μL of diluted toxin in matrix was added to CANARY^®^ Zephyr microcentrifuge tubes and then 10 μL of immunomagnetic capture beads was added. The reaction was placed on a rotator for 30 min at room temperature. At the end of the reaction time, 20 μL of the B-cell biosensor cells (in reconstitution buffer for at least 30 min at room temperature in the dark) was added to the top of the microcentrifuge tubes containing 11 μL (1:5 of the original concentration) of the toxin matrix: immunomagnetic bead complex. Initiation of the Zephyr software will cause the reaction tube to be centrifuged for 5 s before placement into the luminometer to be read immediately. Luminescence was recorded every second for a total of 120 s and displayed live in a graph. At the end of the run, the software uses a proprietary algorithm to determine if the sample was positive or negative. The algorithm that determines positive or negative responses is based on the signal-to-background noise ≥3 and the curve characteristics dependent on up to 28 coefficients that analyze curve characteristics. Each independent experiment was run with duplicates. Detection limits were determined by average of the last two positive read outs for each buffer/matrix from each experiment, *n* = 8 independent experiment for assay buffer and *n* = 6 independent experiment for the milk matrices.

### 4.3. Modified CANARY^®^ Protocol for Acidic Juices: Carrot, Apple, and Orange Juice

The protocol is similar to the one used for assay buffer and milk matrices with the following changes. Acidic juices were spiked individually with BoNT/A complex at various concentrations for 20 min at room temperature. The spiked toxin acidic juices were then neutralized with 5 M Tris pH 8.0 (10% final volume) [19] for 10 min at room temperature before proceeding with the addition of the magnetic capture beads. After the 30 min reaction to allow for binding of toxin: matrix with immunomagnetic capture beads; a magnetic separator was used to capture the complex, removal of the matrix, and replacement with an equal volume of assay buffer. All the subsequent steps following were the same as for Section 4.2 (assay buffer and milk matrix) except 50 μL (the entire reaction) was used with the biosensors.

### 4.4. Assay Protocol for Liquid Egg, Ground Beef, Green Bean Baby Food, and Smoked Salmon

The protocol is similar to Section 4.2 with some modifications. Of note, the BoNT/A complex was spiked directly into each reaction matrix instead of serial dilution as with assay buffer, milks, and acidic juices. Liquid egg (egg yolk and egg white mixed) was diluted 1:10 into assay buffer before the toxin was added into the 1:10 liquid egg/assay buffer mix to give the indicated concentrations. 0.025 g of ground beef (80:20%), green bean baby food, and smoked salmon were added to each tube along with assay buffer and the indicated toxin dose to a volume of 250 μL. The ground beef, green bean baby food, and smoked salmon with toxin were incubated at room temperature for 30 min. The samples were then sheared via pipetting a few times and vortexed vigorously for 1 min before centrifugation at 10,000× *g* for 5 min to pellet the insoluble particulates. 50 μL of the cleared supernatants were then used for the CANARY^®^ reaction with immunomagnetic beads.

### 4.5. Determination of Limits of Detection, Statistical Significance, and PSI Defined Algorithm

The limit of detection was determined to be the average of all of the last two positives samples from each of the independent experiments ± SD determined by the Zephyr program using a proprietary algorithm based upon previous work [32]. The original CANARY assay developed by MIT defined their limit of detection as a value that was greater than three standard deviations above the background signal. Therefore, the maximum relative luminescent unit (RLU) signal from each sample was used as the metric to determine if the sample was negative or positive for the analyte in question. However, there could be potential issues related to only using the maximum RLU signal for a given sample. For example, aberrant spikes could cause an artificially high RLU value resulting in a false positive or, alternatively, a signal with a low signal but discernable curve could yield a false negative. To minimize erroneous results, PSI developed a proprietary mathematical algorithm that analyzes the light output signals from the biosensors to differentiate a true positive curve from true negative background. This analysis was based on a mathematical algorithm first developed by MIT-LL, but that subsequently has been refined at PSI. There are up to 28 different coefficients within this algorithm that may be set to provide filters for the classification of positive and negative assay calls. Examples of these coefficient filters include the amplitude of peak light output, the number of required upslopes in the light output prior to reaching its peak, and the start time of the first of these upslopes. By adjusting these coefficients based upon data from spiked and non-spiked material, the sensitivity and specificity of each assay can be adjusted to achieve the required performance for each analyte and matrix.

## Figures and Tables

**Table 1:** Limits of detection for BoNT/A holotoxin in assay buffer and three milk matrices in the CANARY^®^ assay.

Matrix	Detection Limits (ng/mL)
Assay Buffer	10.0 ± 2.5
Whole Milk	7.4 ± 2.2
2% Milk	7.9 ± 2.5
Non-fat Milk	7.6 ± 2.3

BoNT/A holotoxin was spiked into assay buffer or various milk matrices and serial dilutions were made in order to evaluate the ability of the CANARY^®^ biosensor assay to detect toxin. Samples were determined to be either positive or negative by the Zephyr program using a proprietary algorithm combining traditional signal-to-noise levels as well as up to 28 different coefficients to generate positive or negative curve characteristics. Limits of detection were determined using the results from eight independent experiments in duplicates for assay buffer and six independent experiments with duplicates for the three milk matrices. The final limits of detection (LODs) for each matrix were the average of all of the last two positive read outs per independent experiment ± SD.

**Table 2:** Detection limits of CANARY^®^ biosensor assay in spiked neutralized acidic juices.

Matrix	Detection Limits (ng/mL)
Orange Juice	62.5 ± 21.2
Apple Juice	75.0 ± 21.6
Carrot Juice	32.5 ± 12.0

Acidic juices were first spiked with BoNT/A at various concentrations for 20 min at room temperature and then neutralized with 10% 5 M Tris pH 8.0 for 10 min before the addition of magnetic capture beads and proceeding with the CANARY^®^ biosensor assay. Samples were determined to be either positive or negative by the Zephyr program using a proprietary algorithm. Five independent experiments with duplicates per concentration were evaluated. The detection limit was calculated using the average of the last two positive read-outs for each experiment.

**Table 3:** Detection limits of CANARY^®^ biosensor assay for liquid egg, ground beef, green bean baby food, and smoked salmon.

Matrix	Detection Limits (ng/mL)
Diluted Liquid egg	171.9 ± 64.7
Ground beef	14.8 ± 2.6
Green bean baby food	16.6 ± 6.5
Smoked salmon	62.5 ± 0.0

Liquid egg matrix was diluted 1:10 with assay buffer before toxin was added. Ground beef, green bean baby food, and smoked salmon at 0.025 g were added to assay buffer and toxin was added to a final volume of 250 μL. After incubation for 30 min at room temperature, samples were centrifuged at 10,000× *g* for 5 min. Cleared supernatants were used for the assay. Samples were determined to be either positive or negative by the Zephyr program using a proprietary algorithm. Diluted liquid egg and smoked salmon matrix LOD was from four independent experiments in duplicates. Ground beef LOD was calculated from three independent experiments in duplicates as well as three experiments with a single sample. Green bean baby food’s LOD was calculated from three independent experiments in duplicates and two independent experiments with a single sample. The detection limit was calculated using the average of the last two positive read-outs for each experiment.

## Supplementary Materials

The following are available online at http://www.mdpi.com/2072-6651/10/11/476/s1, Figure S1: CANARY^®^ biosensor assay specifically detects BoNT/A holotoxin in assay buffer but not BoNT/F holotoxin. 0.5 ug in 50 μL (1 mg/mL) was used. One experiment with one reaction per condition was tested, Figure S2: CANARY^®^ biosensor assay specifically detects BoNT/A holotoxin (AHT) in assay buffer and whole milk. BoNT/A holotoxin was tested in 11 μL reaction mixtures with biosensors. Doses were tested in duplicates for each independent experiment, Figure S3: CANARY^®^ biosensor assay specifically detects BoNT/A complex in spiked and then neutralized carrot juice. BoNT/A complex was tested in 50 μL reaction mixtures with biosensors. Doses were tested in duplicates for each independent experiment; Figure S4: CANARY^®^ biosensor assay specifically detects BoNT/A complex in diluted liquid egg and ground beef. BoNT/A complex was tested in 50 μL reaction mixtures with biosensors. Doses were tested in duplicates for each independent experiment; Figure S5: CANARY^®^ biosensor assay specifically detects BoNT/A complex in green bean baby food and smoked salmon. BoNT/A complex was tested in 50 μL reaction mixtures with biosensors. Doses were tested in duplicates for each independent experiment.

## References

[1] Arnon S.S., Schechter R., Inglesby T.V., Henderson D.A., Bartlett J.G., Ascher M.S., Eitzen E., Fine A.D., Hauer J., Layton M. (2001). Botulinum toxin as a biological weapon: Medical and public health management. JAMA.

[2] Center for Disease Control and Prevention (2016). 2015 Annual Report of the Federal Select Agent Program.

[3] Rossetto O., Pirazzini M., Montecucco C. (2014). Botulinum neurotoxins: Genetic, structural and mechanistic insights. Nat. Rev. Microbiol..

[4] Rummel A. (2015). The long journey of botulinum neurotoxins into the synapse. Toxicon.

[5] Tighe A.P., Schiavo G. (2013). Botulinum neurotoxins: Mechanism of action. Toxicon.

[6] Hill K.K., Xie G., Foley B.T., Smith T.J. (2015). Genetic diversity within the botulinum neurotoxin-producing bacteria and their neurotoxins. Toxicon.

[7] Barash J.R., Arnon S.S. (2014). A novel strain of *Clostridium botulinum* that produces type B and type H botulinum toxins. J. Infect. Dis..

[8] Ferreira J.L., Eliasberg S.J., Edmonds P., Harrison M.A. (2004). Comparison of the mouse bioassay and enzyme-linked immunosorbent assay procedures for the detection of type a botulinal toxin in food. J. Food Prot..

[9] Wictome M., Newton K., Jameson K., Hallis B., Dunnigan P., Mackay E., Clarke S., Taylor R., Gaze J., Foster K. (1999). Development of an in vitro bioassay for *Clostridium botulinum* type B neurotoxin in foods that is more sensitive than the mouse bioassay. Appl. Environ. Microbiol..

[10] Pellett S. (2013). Progress in cell based assays for botulinum neurotoxin detection. Curr. Top. Microbiol. Immunol..

[11] Thirunavukkarasu N., Johnson E., Pillai S., Hodge D., Stanker L., Wentz T., Singh B., Venkateswaran K., McNutt P., Adler M. (2018). Botulinum neurotoxin detection methods for public health response and surveillance. Front. Bioeng.Biotechnol..

[12] Sharma S.K., Ferreira J.L., Eblen B.S., Whiting R.C. (2006). Detection of type A, B, E, and F *Clostridium botulinum* neurotoxins in foods by using an amplified enzyme-linked immunosorbent assay with digoxigenin-labeled antibodies. Appl. Environ. Microbiol..

[13] Ching K.H., Lin A., McGarvey J.A., Stanker L.H., Hnasko R. (2012). Rapid and selective detection of botulinum neurotoxin serotype-A and -B with a single immunochromatographic test strip. J. Immunol. Methods.

[14] Babrak L., Lin A., Stanker L.H., McGarvey J., Hnasko R. (2016). Rapid microfluidic assay for the detection of botulinum neurotoxin in animal sera. Toxins.

[15] Koh C.Y., Schaff U.Y., Piccini M.E., Stanker L.H., Cheng L.W., Ravichandran E., Singh B.R., Sommer G.J., Singh A.K. (2015). Centrifugal microfluidic platform for ultrasensitive detection of botulinum toxin. Anal. Chem..

[16] Rasooly R., Stanker L.H., Carter J.M., Do P.M., Cheng L.W., He X., Brandon D.L. (2008). Detection of botulinum neurotoxin-A activity in food by peptide cleavage assay. Int. J. Food Microbiol..

[17] Bagramyan K., Kaplan B.E., Cheng L.W., Strotmeier J., Rummel A., Kalkum M. (2013). Substrates and controls for the quantitative detection of active botulinum neurotoxin in protease-containing samples. Anal. Chem..

[18] Cheng L.W., Stanker L.H., Henderson T.D., Lou J., Marks J.D. (2009). Antibody protection against botulinum neurotoxin intoxication in mice. Infect. Immun..

[19] Cheng L.W., Stanker L.H. (2013). Detection of botulinum neurotoxin serotypes A and B using a chemiluminescent versus electrochemiluminescent immunoassay in food and serum. J. Agric. Food Chem..

[20] Scotcher M.C., Cheng L.W., Stanker L.H. (2010). Detection of botulinum neurotoxin serotype B at sub mouse LD(50) levels by a sandwich immunoassay and its application to toxin detection in milk. PLoS ONE.

[21] Stanker L.H., Merrill P., Scotcher M.C., Cheng L.W. (2008). Development and partial characterization of high-affinity monoclonal antibodies for botulinum toxin type A and their use in analysis of milk by sandwich elisa. J. Immunol. Methods.

[22] Stanker L.H., Scotcher M.C., Cheng L., Ching K., McGarvey J., Hodge D., Hnasko R. (2013). A monoclonal antibody based capture elisa for botulinum neurotoxin serotype B: Toxin detection in food. Toxins.

[23] Bagramyan K., Barash J.R., Arnon S.S., Kalkum M. (2008). Attomolar detection of botulinum toxin type A in complex biological matrices. PLoS ONE.

[24] Bagramyan K., Kalkum M. (2011). Ultrasensitive detection of botulinum neurotoxins and anthrax lethal factor in biological samples by alissa. Methods Mol. Biol..

[25] Barr J.R., Moura H., Boyer A.E., Woolfitt A.R., Kalb S.R., Pavlopoulos A., McWilliams L.G., Schmidt J.G., Martinez R.A., Ashley D.L. (2005). Botulinum neurotoxin detection and differentiation by mass spectrometry. Emerg. Infect. Dis..

[26] Wang D., Baudys J., Hoyt K.M., Barr J.R., Kalb S.R. (2017). Further optimization of peptide substrate enhanced assay performance for BoNT/A detection by MALDI-TOF mass spectrometry. Anal. Bioanal. Chem..

[27] Kalb S.R., Baudys J., Wang D., Barr J.R. (2015). Recommended mass spectrometry-based strategies to identify botulinum neurotoxin-containing samples. Toxins.

[28] Liu J., Gao S., Kang L., Ji B., Xin W., Kang J., Li P., Gao J., Wang H., Wang J. (2017). An ultrasensitive gold nanoparticle-based lateral flow test for the detection of active botulinum neurotoxin type A. Nanoscale Res. Lett..

[29] Orlov A.V., Znoyko S.L., Cherkasov V.R., Nikitin M.P., Nikitin P.I. (2016). Multiplex biosensing based on highly sensitive magnetic nanolabel quantification: Rapid detection of botulinum neurotoxins A, B, and E in liquids. Anal. Chem..

[30] Dunning F.M., Ruge D.R., Piazza T.M., Stanker L.H., Zeytin F.N., Tucker W.C. (2012). Detection of botulinum neurotoxin serotype A, B, and F proteolytic activity in complex matrices with picomolar to femtomolar sensitivity. Appl. Environ. Microbiol..

[31] Worbs S., Fiebig U., Zeleny R., Schimmel H., Rummel A., Luginbuhl W., Dorner B.G. (2015). Qualitative and quantitative detection of botulinum neurotoxins from complex matrices: Results of the first international proficiency test. Toxins.

[32] Rider T.H., Petrovick M.S., Nargi F.E., Harper J.D., Schwoebel E.D., Mathews R.H., Blanchard D.J., Bortolin L.T., Young A.M., Chen J. (2003). A B cell-based sensor for rapid identification of pathogens. Science.

[33] Bartholomew R.A., Ozanich R.M., Arce J.S., Engelmann H.E., Heredia-Langner A., Hofstad B.A., Hutchison J.R., Jarman K., Melville A.M., Victry K.D. (2017). Evaluation of immunoassays and general biological indicator tests for field screening of *Bacillus anthracis* and ricin. Health Secur..

[34] Center for Food Safety and Applied Nutrition, U.S. Food and Drug adminstration (FDA) (2001). Bacteriological Analytical Manual.

[35] Maslanka S.E., Solomon H.M., Sharma S., Johnson E.A., Doores S., Salfinger Y., Tortorello M.L. (2013). Clostridium botulinum and its toxins. Compendium of Methods for the Microbiologial Examination of Foods.

[36] Kalluri P., Crowe C., Reller M., Gaul L., Hayslett J., Barth S., Eliasberg S., Ferreira J., Holt K., Bengston S. (2003). An outbreak of foodborne botulism associated with food sold at a salvage store in Texas. Clin. Infect. Dis..

[37] Angulo F.J., Getz J., Taylor J.P., Hendricks K.A., Hatheway C.L., Barth S.S., Solomon H.M., Larson A.E., Johnson E.A., Nickey L.N. (1998). A large outbreak of botulism: The hazardous baked potato. J. Infect. Dis..

[38] Juliao P.C., Maslanka S., Dykes J., Gaul L., Bagdure S., Granzow-Kibiger L., Salehi E., Zink D., Neligan R.P., Barton-Behravesh C. (2013). National outbreak of type A foodborne botulism associated with a widely distributed commercially canned hot dog chili sauce. Clin. Infect. Dis..

[39] Sheth A.N., Wiersma P., Atrubin D., Dubey V., Zink D., Skinner G., Doerr F., Juliao P., Gonzalez G., Burnett C. (2008). International outbreak of severe botulism with prolonged toxemia caused by commercial carrot juice. Clin. Infect. Dis..

