# Supplementary material for: A Rapid, Sensitive, and Portable Biosensor Assay for the Detection of Botulinum Neurotoxin Serotype A in Complex Food Matrices
Source: Toxins (Basel). 2018 Nov 15;10(11):476. doi: 10.3390/toxins10110476 (PMC6266793; doi:10.3390/toxins10110476)
Supplement: Supplementary file 1 [file toxins-10-00476-s001.pdf]

Supplementary Materials: A Rapid, Sensitive, and Portable Biosensor Assay for the Detection of Botulinum Neurotoxin Serotype A in Complex Food Matrices

Christina C. Tam, Andrew R. Flannery and Luisa W. Cheng

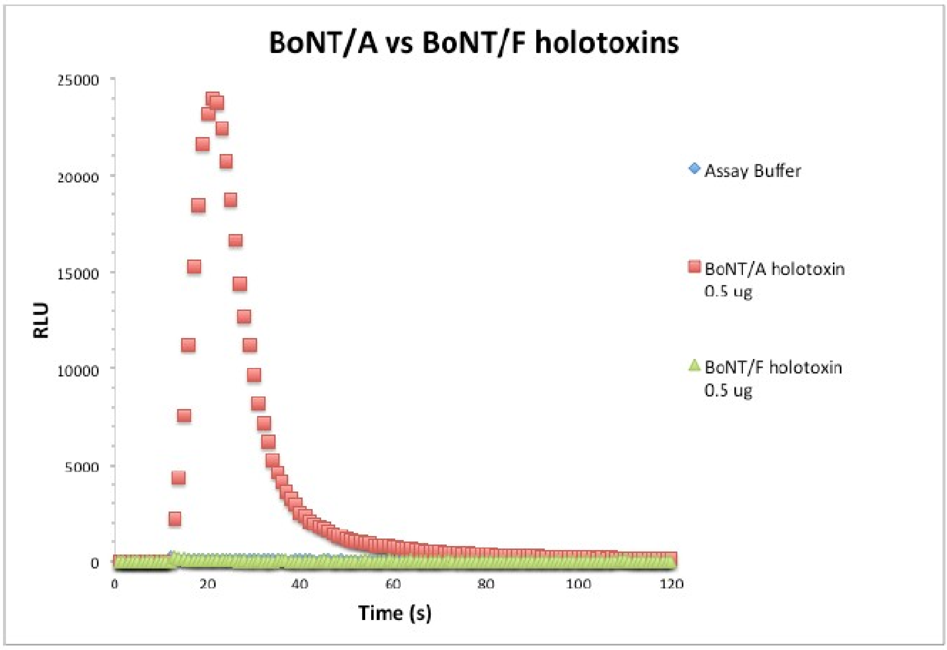

Figure S1. CANARY® biosensor assay specifically detects BoNT/A holotoxin in assay buffer but not BoNT/F holotoxin. 0.5 ug in 50 µL (1 mg/mL) was used. One experiment with one reaction per condition was tested.

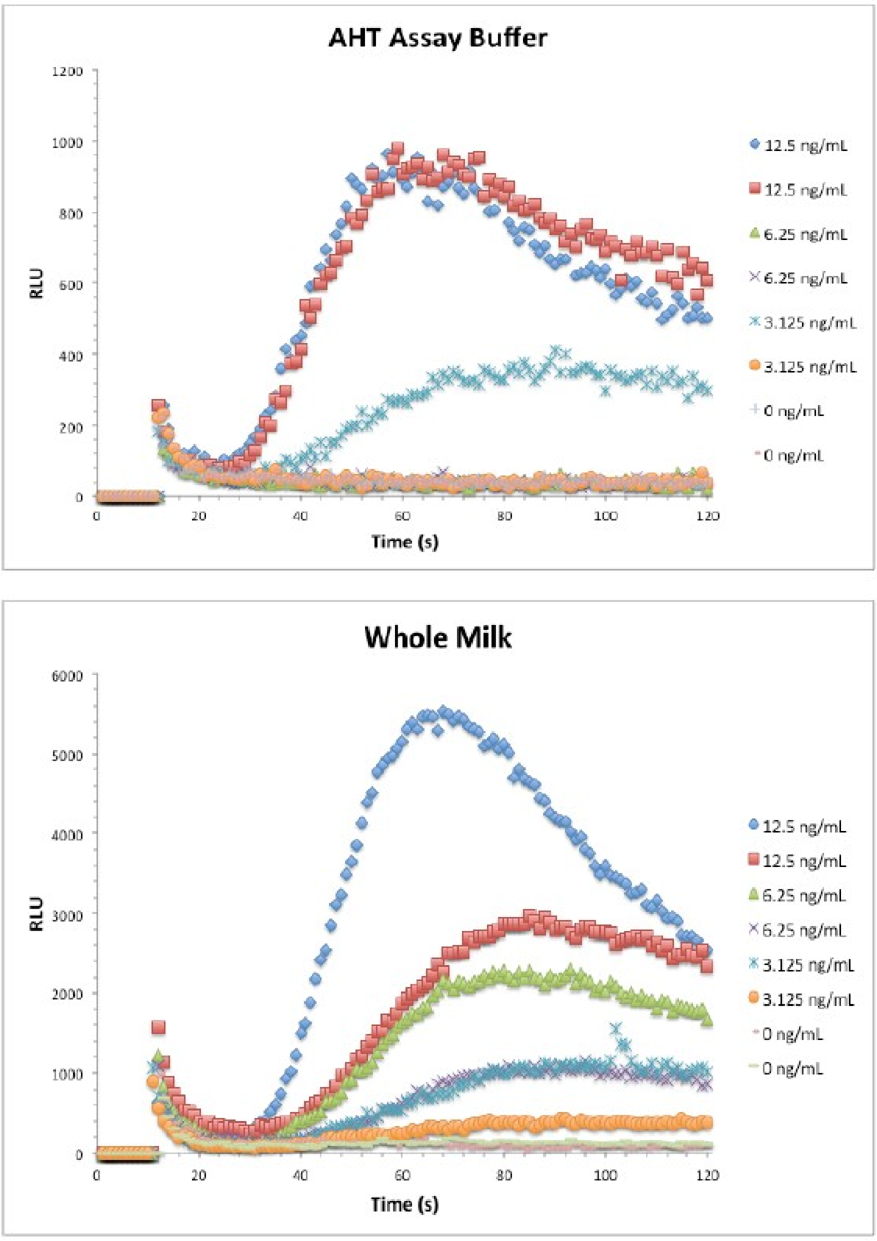

Figure S2. CANARY® biosensor assay specifically detects BoNT/A holotoxin (AHT) in assay buffer and whole milk. BoNT/A holotoxin was tested in 11 µL reaction mixtures with biosensors. Doses were tested in duplicates for each independent experiment.

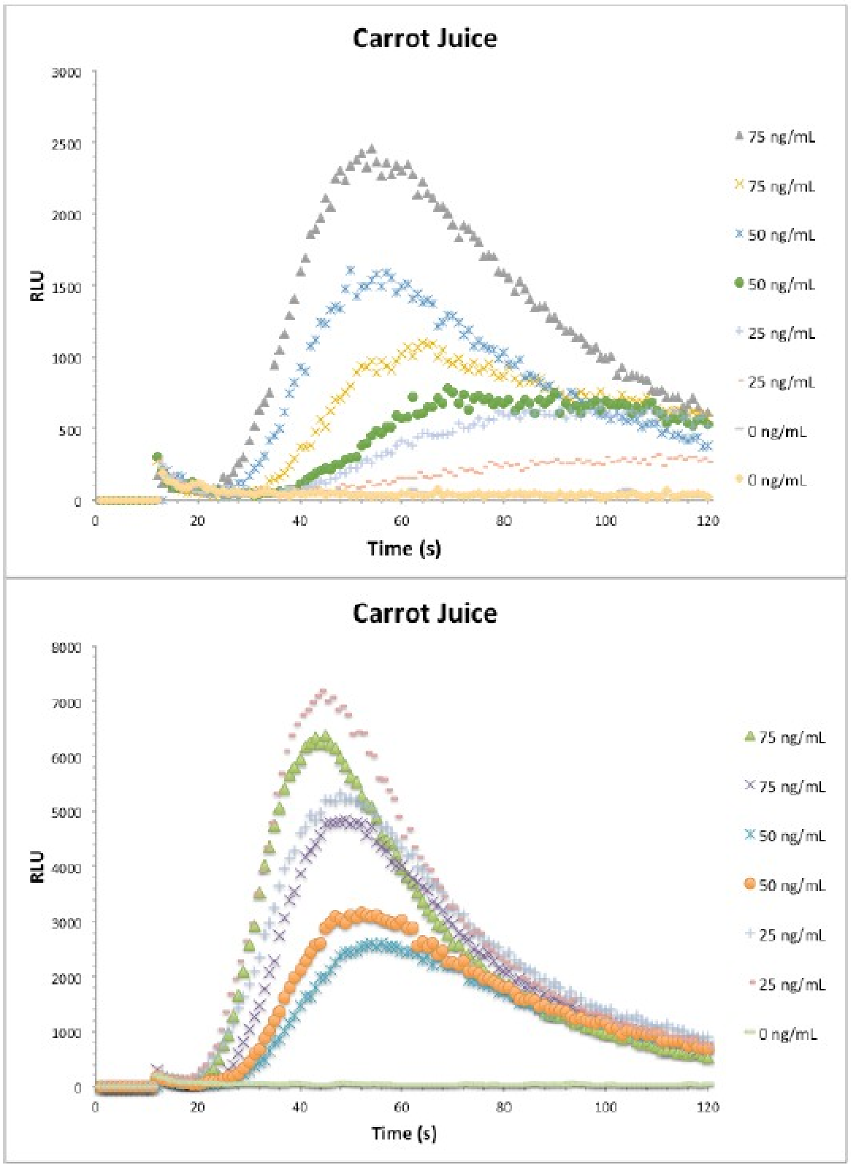

Figure S3. CANARY® biosensor assay specifically detects BoNT/A complex in spiked and then neutralized carrot juice. BoNT/A complex was tested in 50 μL reaction mixtures with biosensors. Doses were tested in duplicates for each independent experiment.

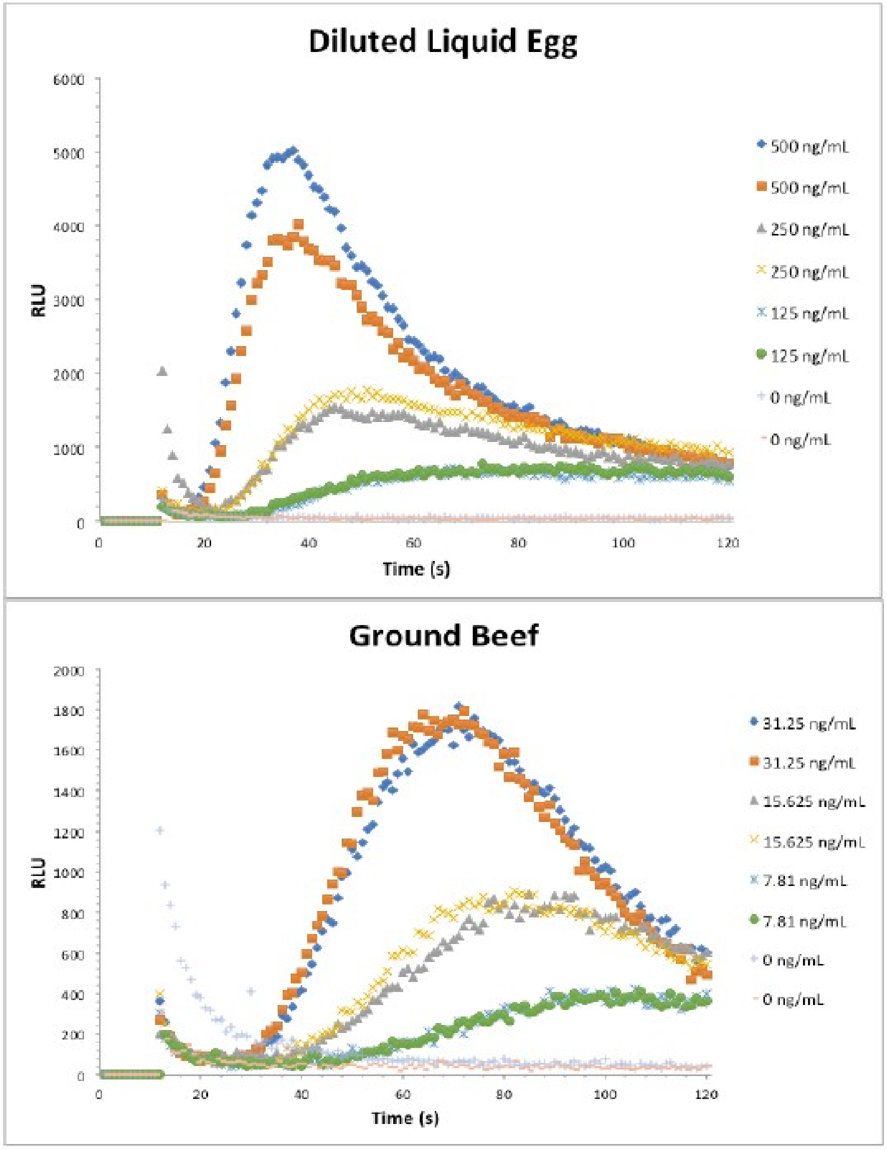

Figure S4. CANARY® biosensor assay specifically detects BoNT/A complex in diluted liquid egg and ground beef. BoNT/A complex was tested in 50 μL reaction mixtures with biosensors. Doses were tested in duplicates for each independent experiment.

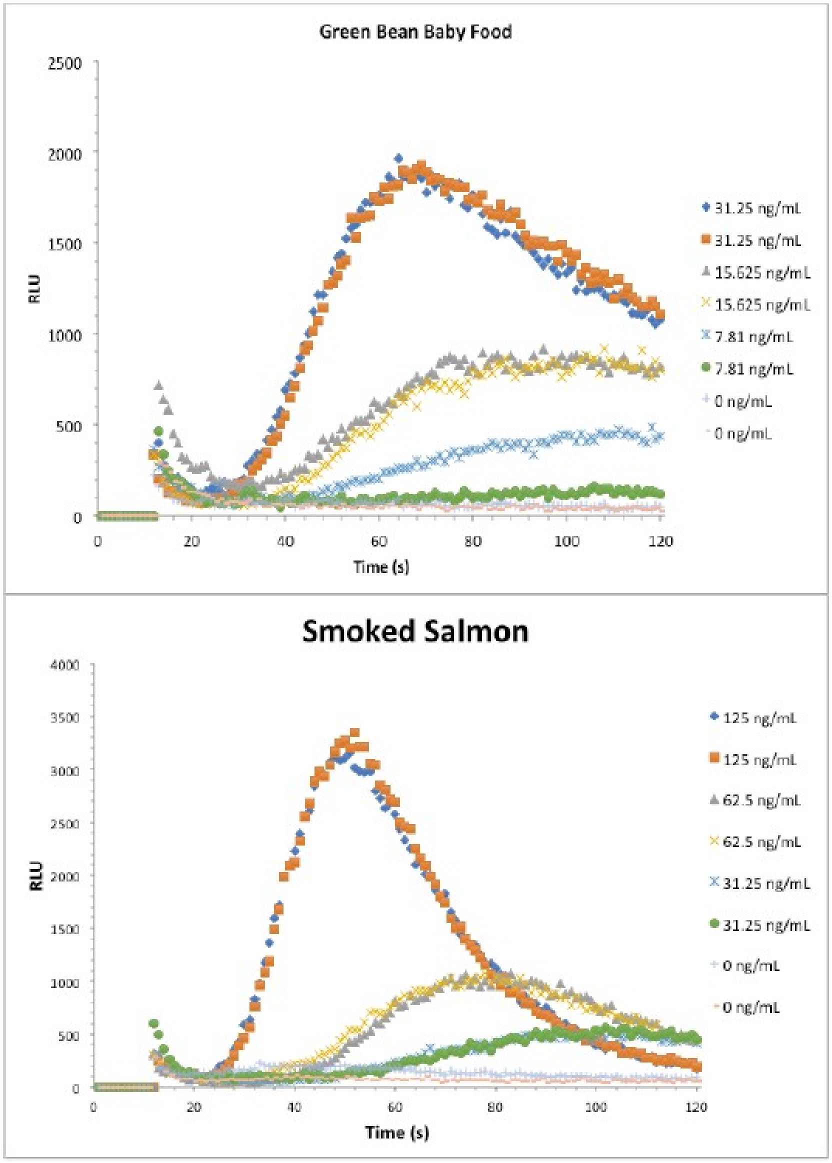

Figure S5. CANARY® biosensor assay specifically detects BoNT/A complex in green bean baby food and smoked salmon. BoNT/A complex was tested in 50 μL reaction mixtures with biosensors. Doses were tested in duplicates for each independent experiment.
